# Spatial nonlinear optics for securing information

**DOI:** 10.1038/s41377-021-00699-z

**Published:** 2022-02-07

**Authors:** Wen Chen

**Affiliations:** Department of Electronic and Information Engineering, The Hong Kong Polytechnic University, Hong Kong, China

**Keywords:** Applied optics, Optical techniques

## Abstract

The high degrees of freedom of light, various optical structures and optical materials can be explored and applied to develop optical encryption for securing information. An exciting optical image encryption approach has been proposed based on spatial nonlinear optics.

With a rapid development of modern technologies, it has been well recognized that information security plays an important role in many applications, e.g., secured data storage and communication. Securing information with optical means has attracted much current attention, since double random phase encoding^1^ was proposed. The remarkable characteristics of optical encryption^2–9^, e.g., parallel processing and multi-dimensional capabilities, have been continuously explored. For instance, parallel processing can be realized by using optical devices, and multiple-dimensional and multiple-parameter capabilities of the light are explored and applied in optical image encryption systems.

Various transform domains, optical materials and optical imaging methods^2–9^ have been studied for optical encryption, e.g., digital holography, computer-generated hologram, diffractive imaging, and ghost imaging. These designs, infrastructures and algorithms have greatly enriched optical image encryption field, and make optical encryption to be feasible and effective in practical applications. However, it has also been demonstrated and verified that linearly optical image encryption systems may be attacked, and security vulnerability could be detected by using some properly-designed algorithms^10,11^, e.g., chosen-ciphertext attack and known-plaintext attack. Therefore, the breakthroughs need to be continuously made in the field of optical encryption to achieve high security and high applicability.

To overcome the challenges, Hou and Situ^12^ proposed to apply spatial nonlinear optics for securing information. Nonlinear characteristic is obtained for optical image encryption in an optical setup using Sr_0.61_Ba_0.39_Nb_2_O_6_ (SBN:61) crystal^12^ as schematically shown in Fig. 1. To encode an image, two statistically-independent random phase patterns are embedded into phase-only spatial light modulators, and the nonlinearity is generated by using photorefractive crystal in the designed optical encoding setup. The nonlinear Schrӧdinger transform has been defined and applied in the proposed optical encryption approach^12^.

**Fig. 1:**
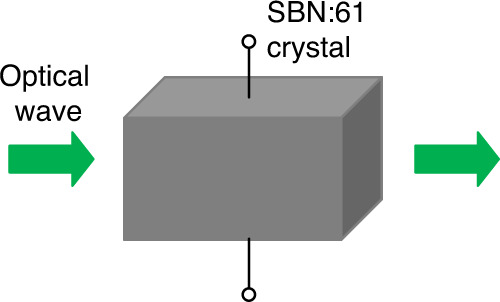
System nonlinearity. Schematic of the nonlinearity generated by using photorefractive crystal in optical encryption

Regarding misalignment toleration, robustness against noise and effect of nonlinearity strength, the analysis has been conducted in detail to show good performance of the proposed nonlinear encryption approach^12^. It is also demonstrated that owing to self-phase modulation effect of photorefractive crystal, the proposed approach is robust against known-plaintext attack. In addition, the proposed nonlinear encryption approach can withstand deep learning-based attacks, since plaintext-dependent strategy is integrated into the designed optical encryption system. The work in Ref. ^12^ sheds light on optical information security based on spatial nonlinear optics.

In optical encryption, there are still some significant challenges that deserve more attention and effort to overcome, and high encoding capacity, high applicability and high security are desired. Various optical parameters, optical structures and optical materials, e.g., metasurface, can be continuously designed and applied to realize high-capacity and high-security nonlinearly optical encryption. With the development of optical instruments and nanomaterials, it can be expected that high-speed data decoding using an optical way could also be feasible. The continuously-developed new concepts can create a new era of optical encryption.
